# Heat in the Treatment of Cerebro-Spinal Meningitis

**Published:** 1876-08

**Authors:** 


### Selections.

---

### HEAT IN THE TREATMENT OF CEREBRO-SPINAL MENINGITIS.

Dr. J. D. Bennett, in *The Clinic,* thus remarks upon the treatment of *Cerebro-Spinal Meningitis:* This terrible disease seems more common than formerly, and a malady of so fearful a nature and such frightful mortality, that it certainly demands every item of information we can glean.

It prevailed in many parts of the country in the fall and and winter of '72 and '73. In our vicinity, Assumption, Illinois, it became an epidemic in November, and continued through the winter. That season we were visited by no special epidemic, except chills and fever, which are not uncommon in Illinois. There were some cases of erysipelas in the country; but I could see no connection between the two diseases. It never seemed contagious, though more than one case was sometimes in a family; if there were any special or local causes to produce it, the physicians failed to find them. It occurred alike in the families of rich and poor; two-thirds of the cases were children.

The symptoms are so fully described in the text-books that it would be a work of supererogation on my part to go into a description of them.

*Treatment.*—This is a much vexed question, and as far as I can learn, no special treatment is very successful. Dr. Smith, in his excellent article on "Cerebro-Spinal Fever" in the *American Journal* for October, 1873, says: In the best conducted treatment in the epidemic of that year in New York, one-half died. This is far above the general average; in many places the mortality is much above one-half. The course I have found most successful was large doses of bromide of potassium repeated every hour or two, a blister over the spine, cold applications to the head and the *continued application of heat to the cutaneous surface.* I regard this last of more importance than any one other thing in the treatment. Dr. Smith, in this article, says: A hot mustard foot bath or general warm bath with mustard should be employed as early as possible, since it acts so powerfully as a derivative from the hyperæmic nerve centres, tends to calm the nervous excitement and prevent convulsions. Now if Dr. S. had to remain all night with his patients, as country practitioners sometimes do, he would have found that hot bricks or bottles act as powerfully as derivatives from the hyperæmic nerve centres as a hot bath, and even more, as you cannot long continue a hot bath

Continued heat to the skin greatly relieves the suffering, the skin becomes less spotted, and it relieves the opisthotonos, and helps in every way. Again, Dr. Smith says: The internal temperature is always increased and temperature of the cutaneous surface is frequently below that of the blood. Here is another indication for hot applications. Long before I saw these statements I found the temperature of the surface was abnormally low, and to increase the coldness was to increase the pain; this we can do by opening a door or uncovering an arm or leg. The skin may at first present a natural appearance, but soon assumes the peculiar "goose flesh," then the dark mottled or spotted appearance, something like faded calico, and the patient's suffering is sure to increase. In a few cases these spots are not present. Rubbing the skin would seem to be indicated, but this the irritation it produces forbids. My usual way of applying heat is after the hot bath, fill a small jug with hot water, wrap it with flannel and apply to the feet, fill some bottles and apply to the legs and hips, and keep them there as long as they afford *relief*, for which the patient is too glad to be much annoyed by their presence.

There is one thing should be remembered, both the internal and external temperatures are subject to very sudden variations. These correspond with the fluctuations of the pulse which rises or falls according as the internal temperature varies, and we have to regulate our treatment as these changes occur, gradually abating the hot applications as the pulse becomes normal.

Bromide of potassium, according to Hammond, produces a sedative effect due to cerebral anæmia brought about by contracting the arterioles of the brain, and it is a powerful and safe nervous sedative; it could scarcely fail to be an excellent remedy, and, as far as it goes, there is none better. I used it in large doses in every case. Hydrate of chloral is also a useful sedative. Opium is praised by some, and, of course, will relieve the suffering, but I am satisfied it tends to bring on coma, which we so much dread. If in this disease a physician will give sufficient morphine to quiet the patient, and watch the frightful similarity between stupor from morphine and coma from cerebro-spinal meningitis, it ought to make him very cautious about pushing that remedy. Yet, *small* doses of morphine combined with the bromide will very much relieve the intense snffering. Cold water to the head is very useful, it relieves the congestion of the brain by contracting the blood vessels. I gave ergot in almost every case, but have no great confidence in it as a remedy. Quinine, as far as my experience goes, does not the least good. After the severe symptoms have passed, I gradually left off the bromide and gave the iodide of potash with the hope of absorbing the effused fluids of the brain. To relieve the severe nervous prostration following the disease I know of nothing better than strychnine combined with tinct. of iron; at all times giving the patients as much nutritious food as they could take; give a little whisky toward the last.

Case.—J. W., blacksmith, had been unwell for three or four days, somewhat exhausted watching a sick relative. March 18th. Taken with violent pains in the head, spine and limbs; in fact, "pain all over," as he expressed it. Saw him in a short time, he was unconscious, lying on the floor with head and shoulders drawn back to one-third circle, violent action of the extensors of arms and legs, convulsive jerking of the face, pupils dilated, did not respond to light; pulse quick and irregular, breathing heavy and labored. Tried several sedatives

(opium, spts. ether, etc.), but none seemed to relieve him; then gave chloroform by inhalation, which relieved him as long as the anæsthetic effect lasted, then he was as bad as ever. Repeated the chloroform till the third time, when I had him wrapped in hot blankets and applied the warm bottles; he remained comparatively quiet, but would occasionally groan; slight opisthotonos continued; gave the bromide. Returning ten hours afterwards found considerable opisthotonos and head drawn to the right; skin cold and spotted; pulse 98. Still, his symptoms were all better than the previous day. The attendants had given the sedative, but removed the hot bottles. I immediately replaced them, when he seemed better. March 21st. Pulse 92, pupils a little dilated, considerable pain and head drawn backward and a little to the right. The warm application had been removed four hours before and he had been getting worse; I again replaced them when he became easier. By this time he was entirely conscious and remained so. I neglected to say I gave purgatives from the first sufficient to keep his bowels open, and the first day put a blister to his spine. Combined hydrate of chloral with the bromide and kept up the cold cloths to his head. Left strict orders with his brother and sister to keep up the warm applications, which they faithfully did, not letting him again get cold. Gradually left off the bromide, and gave the iodide of potash; then gave strychnine and iron. On the sixth day he was able to sit up in bed, but still had shooting pains through the head and spine, which gradually subsided. The main feature in the case was the relief he obtained from the *hot applications*. At three different times he seemed more relieved by them than had he taken a large dose of morphine, and the last time the severe symptoms never returned, as they were not entirely removed for four days, until he began to recover.

I had three cases in the beginning of the epidemic which I treated with quinine, morphine, blister, and cold to the head, and bromide of potassium, and all three died. After changing the treatment, I had six well-marked cases, and the last one a young man who had just recovered from a severe attack of erysipelas. This does not include every one who had pain in the head and spine, as many were quite sick for two or three days with several severe symptoms of the fever, and I am satisfied it was a modified form of the same complaint. This is common in this disease. I saw several of Dr. Herdman's patients at the same time, and he treated them in the same way, and with equal success, and he had more cases than I. He and I began this course at the same time—that is, the continued application of dry heat to the cutaneous surface. We were led to this by watching a severe case in Dr. H.'s family—a little girl who was very ill. We noticed when the room was cold, or the cover was removed, she would begin to scream and be very restless, the skin became cold and dark spotted, and she would become better when warm. Thinking over the case, we concluded to try it in our practice. There were six other cases in the community outside our practice, and all died. These were all treated differently.

I know a few cases in a single epidemic is not a sufficient test to establish any treatment, but dry heat was certainly of great benefit in our hands.—*The Clinic.*

